# Marital Quality and Smoking among Couples in Mid- and Later-Life

**DOI:** 10.1093/geroni/igaf122.2935

**Published:** 2025-12-31

**Authors:** Angela Curl, Amy Roberts, Jennifer Bulanda

**Affiliations:** Miami University, Oxford, Ohio, United States; Miami University, Oxford, Ohio, United States; Miami University, Oxford, Ohio, United States

## Abstract

This study investigates the relationship between smoking and marital quality in older married couples, focusing on how marital dynamics influence smoking behaviors in later life. We hypothesized that both own and spouse’s ratings of negative marital quality would increase the likelihood of smoking as a coping strategy (Smith & Gibson, 2019). Using pooled data (N = 1,239 individuals) from the 2014-2018 waves of the Health and Retirement Study, we examined smoking status in a sample of older married couples who had reported being smokers at some point in their lives. Actor-Partner Interdependence Models (APIM) were analyzed with HLM 6.08 software. Marital quality was measured using a 3-item positive scale and a 4-item negative scale, controlling for baseline smoking status. Contrary to expectations, higher negative marital quality in husbands was associated with decreased odds of smoking at Wave 2 (OR = 0.33, p<.05), while positive marital quality was not significant for either partner, and wives’ negative marital quality was also not significant. The strongest predictor of smoking was baseline smoker status, with husbands showing an odds ratio of 137.28 and wives an odds ratio of 896.11 (p<.01). Our findings of negative marital quality being associated with better health outcomes are consistent with Liu, Shen, and Waite (2016), who found that negative marital quality lowered the risk of developing diabetes and increased diabetes management. These findings highlight the persistent long-term influence of past smoking behaviors over current marital quality in shaping smoking patterns in later life.

